# Pycnogenol® Supplementation Prevents Recurrent Urinary Tract Infections/Inflammation and Interstitial Cystitis

**DOI:** 10.1155/2021/9976299

**Published:** 2021-06-23

**Authors:** A. Ledda, S. Hu, M. R. Cesarone, G. Belcaro, M. Dugall, B. Feragalli, R. Cotellese, M. Hosoi, E. Ippolito, M. Corsi, R. Luzzi

**Affiliations:** IRVINE3 Vascular/Circulation Labs CH-PE University, Pescara, Italy

## Abstract

This open pilot registry study aimed to evaluate and compare the prophylactic effects of Pycnogenol® or cranberry extract in subjects with previous, recurrent urinary tract infections (UTI) or interstitial cystitis (IC). *Methods*. Inclusion criteria were recurrent UTI or IC. One subject group was supplemented with 150 mg/day Pycnogenol®, another with 400 mg/day cranberry extract, and a group served as a control in a 2-month open follow-up. *Results*. 64 subjects with recurrent UTI/IC completed the study. The 3 groups of subjects were comparable at baseline. All subjects had significant symptoms (minor pain, stranguria, repeated need for urination, and lower, anterior abdominal pain) at inclusion. In the course of the study, the subjects reported no tolerability problems or side effects. The incidence of UTI symptoms, in comparison with the period before inclusion in the standard management (SM) group, decreased significantly; there was a more pronounced decrease in the rate of recurrent infections in the Pycnogenol® group (*p* < 0.05). The improvement in patients supplemented with Pycnogenol® was significantly superior to the effects of cranberry. At the end of the study, all subjects in the Pycnogenol® group were infection-free (*p* < 0.05vs. cranberry). Significantly, more subjects were completely symptom-free after 2 months of management with Pycnogenol® (20/22) than with SM (18/22) and cranberry (16/20). *Conclusions*. This pilot registry suggests that 60 days of Pycnogenol® supplementation possibly decrease the occurrence of UTIs and IC without side effects and with an efficacy superior to cranberry.

## 1. Introduction

Urinary tract infections (UTIs) are common clinical observations. Roughly, 50% of women worldwide contract at least one UTI in their life and about 25–30% of women have at least one episode of recurrent urinary infection [1, 2]. UTIs in men are also common and often recurrent in subjects with lower urinary tract anomalies or anatomical variations [2]. Patients suffering from partial or temporary urinary tract obstruction after catheterization or surgery and subjects with benign prostatic hypertrophy are also often affected by UTI [3, 4]. Especially in elderly patients, UTIs are often overlooked or misdiagnosed due to problems with the interpretation of the urinalysis [5].

Recurrent UTIs (R-UTI) are defined as a sequence of “three episodes in the previous year or two episodes in the last six months” [6, 7]. Furthermore, R-UTIs can be described as mild/moderate intensity when signs or symptoms last less than 3 days and no hospitalization is needed.

The American Urological Association and the Infectious Disease Society of America recommend applying treatment with nitrofurantoin for 5 days or one dosage of fosfomycin (Monuril) as a primary antibiotic treatment of acute cystitis [1, 7]. Alternative antibiotics are used according to local practice [1, 7]. Longer-term antibiotic prophylaxis may also be used in subjects with more complex R-UTIs [1, 6, 7]. But frequent or long-term use of antibiotics increases costs and side effects and may select multidrug resistant organisms. Prolonged use of nitrofurantoin has been linked to anemia, pulmonary toxicity, hepatic disease, and neuropathy [1, 8]. Currently, a standard recommendation for prophylactic antibiotic treatment for R-UTI is not available [1, 9, 10]. Symptoms and inflammation can continue even without present bacteria or with only a small bacterial charge in the urine after the acute phases, as a lower tract, urinary infection may have a significant concomitant, inflammatory, nonbacterial component [11–13]. The inflamed urothelial cells may cause signs and symptoms of UTI and may also attract other bacteria, restarting infections [14]. In many subjects, symptoms of UTI may be an expression of interstitial cystitis, difficult to diagnose and manage [14].

Interstitial cystitis (IC) is a painful, often chronic disorder of the bladder, typically indicated by serious sensory lower urinary tract symptoms and pelvic pain [15, 16]. IC is not caused by bacteria or viruses, but instead, it is described as having different potential etiologies, such as autoimmune processes, vascular occlusions, hormonal or psychogenic factors [16, 17]. The estimated prevalence of IC is reported to be between 3 and 6% in adult women [14].

Recently, effective standardized supplements (i.e., cranberry (*Vaccinium macrocarpon Ait.)* and Pycnogenol® (extract from *Pinus pinaster Ait.*)) have been tested as prophylactic agents to prevent R-UTIs in subjects at risk of developing recurrent lower urinary tract infections [9, 11, 18, 19]. Cranberries have been used for UTI in various dosage forms in prevention and treatment studies [1, 9, 11].

Burleigh et al. have evaluated the effects of cranberry preparation on UTI and R-UTI incidences in women [9]. After 6 months of cranberry consumption, the number of UTIs was reduced [9]. Regarding the extract from French maritime pine bark (Pycnogenol®), components present in this supplement have been associated with antimicrobial and bacteriostatic activity in several studies [20–24].

This open pilot registry was conducted to evaluate and compare any prophylactic effects of oral supplementation with Pycnogenol® or with cranberry in subjects with a previous, recent history of R-UTI or IC in a follow-up for 2 months. In addition, the antioxidative and the anti-inflammatory effects of both compounds on nonbacterial interstitial cystitis were investigated.

## 2. Patients and Methods

### 2.1. Inclusion and Exclusion of Subjects

Subjects with a recent (<12 months) history of recurrent-UTI/IC were included if

- They reported at least three symptomatic UTIs in the past year
- They reported at least two UTIs in the past six months
- They reported symptoms of UTIs without bacterial occurrence in the urinalysis, as in IC

Exclusion criteria included diabetes, any other chronic clinical condition or risk conditions, immune-compromising diseases, comorbidities, antibiotic or corticosteroids treatment for any other reason, mycosis, use of chemotherapy within 6 months before inclusion, chronic inflammatory bowel disease, and any possible or suspected intolerance or allergy to the supplements. In the case of visible blood in the urine of the patients, they were not included in the registry.

According to common clinical practice [1], subjects with a history of minor symptoms of a single, initial episode of UTI received a single dose of the antibiotic fosfomycin (Monuril) and were included in the registry study. In case of more severe symptoms or recurrent-UTI (R-UTI), patients were treated with two doses in two days but were not included in the registry study. Subjects with urinary tract infections using other antibiotic treatments were also excluded from the study to have a homogeneous and comparable registry population.

### 2.2. Supplementation

At 5 days, if a urinalysis indicated the absence of blood or a clinically significant bacterial charge (according to the upper limits in Table 1), the patients took Pycnogenol® (150 mg/day) or cranberry extract (400 mg/day) for 60 days.

Studies report about the effects of cranberry (*Vaccinium macrocarpon Ait*.) in R-UTIs [9, 11], but a standard regimen has not been defined since there is no standardized cranberry product. An intake of 36 mg proanthocyanidins per day has been shown to contribute to decreasing UTIs and inflammation of the urinary tract [25]. As suggested in a review by M. Hisano and colleagues, two capsules per day containing 200 mg of dried cranberry extract (19 mg proanthocyanidins each) were used, providing the necessary amount of daily proanthocyanidins for decreasing UTIs [25]. Pycnogenol® (Horphag Research) is a highly standardized extract from the bark of the French maritime pine (*Pinus pinaster* Ait.), consisting of a concentrate of polyphenols, standardized to 65–75% procyanidin content with antioxidant, and anti-inflammatory activities [26, 27]. The (pre)clinically beneficial role of Pycnogenol® in reducing the level of inflammation was confirmed in several clinical studies [26,27].

### 2.3. Subjects

64 subjects between 35 and 41 years old completed the registry study (Table 2). The occurrence of new episodes in a two-month follow-up was evaluated. Any new episode was indicated by described signs and symptoms, presence of increased bacterial charge (assessed by urinalysis), visible blood in the urine, or need for a medical appointment.

An associated “standard management” (SM) was used by all subjects. Accurate hygiene (without using local disinfectants), improved bladder care (with drinking and voiding at an appropriate time, according to needs), avoiding too much caffeine, spices, and alcohol, and careful hydration was suggested. A program of mild exercise was also implemented (20–30 minutes walking daily, avoiding sitting in the same position for hours).

### 2.4. Target Parameters

In this registry study, the clinical efficacy of the supplement was assessed by three target parameters:

1. The number of UTIs in the subjects were compared between the two periods, 2 months before and 2 months after inclusion
2. The number of subjects without infection at the end of the registry
3. The number of normal values of the respective parameters (see Table 1[13]) in the urinalysis at the end of the study

The number of infection-free subjects was confirmed by assessment of the urine in urinalysis and by the absence of signs/symptoms for 2 months.

Systemic oxidative stress was assessed by the measurement of the plasma free radical concentration, as previously described [28]. In short, the reactive oxygen metabolites-derived compounds (d-ROMs) were measured, determining the plasmatic hydroperoxides levels. 0.15–0.2 mL of blood from the finger of fasting patients were taken. After plasma isolation by centrifugation, the levels of hydroperoxides in the plasma were quantified and the result was given in Carr units.

### 2.5. Characteristics of This Registry and Follow-Up

The present study is an independent, open, small-scale, pilot registry study. The substances of interest were only recommended and not prescribed. Instead of selecting groups of subjects with certain conditions that often do not correspond to the clinical reality, this type of study is closer to the practical application and may be better suited for developing countries and in the circumstances without big sponsorships. An independent board not in contact with the subjects of the study analyzed and compared the data of this registry.

During the 2 months follow-up, subjects were asked to report any new signs/symptoms of UTI/IC and to follow the standard management. Throughout the follow-up time, weekly contacts and laboratory measurements were conducted to assess any issues regarding safety, tolerability, and compliance. During the registry, any adverse effect was recorded and evaluated, with reference to duration, intensity, outcome, and potential connection to the study products.

### 2.6. Supplement Studies

The present type of study is designed to characterize potential fields of application of (preferably standardized) supplements for the preventive, nonclinical use in subjects with borderline risk conditions [29–35]. The use of supplements is generally not intended for the treatment of symptoms in clinical disorders. The planning and organization of these studies require active participation and the full attention of the study subjects. Usually, the procedure of these studies is to collect data, which is compared to different management plans or to historical data, with the recommended treatment of comparable subjects.

In the present study, the supplements were used as follows:

1. The admission of the supplements was suggested to the participating subjects without prescription as a possible option, which might improve risk condition leading to recurrent UTI.
2. The patients were treated according to the recommended standard management for UTI, R-UTI, or IC and the supplements were only used on top of international guidelines.
3. Any interference of the supplements with standard treatment or other preventative measures for UTI, R-UTI, or IC would have been an exclusion criterium.
4. The follow-up time is variable and was adapted to the availability of the registry subjects and until positive changes were noticed.
5. The evaluation type of studies like the present is a registry.
6. The supplements are freely available without a prescription and are self-acquired by the study subjects. Underprivileged subjects can profit from a freely available quantity of products. By assessing the compliance for taking the dietary supplements, the desire to take the products becomes clear, which is an interesting and significant value for medicinal practice.
7. There is no randomization and no group assignment coordinated by the investigators in supplement studies.
8. After the initial briefing, subjects decide which management group they want to enter including the control group, in which subjects are not taking any supplements or placebo and which is not necessarily parallel.
9. This study is an open-label study, which means that subjects are well informed about any supplements, treatments, and management measures they are receiving. The possibility of a placebo effect is considered and carefully explained.

All resulting data is analyzed after the follow-up period when sufficient evidence has been gathered.

### 2.7. Statistical Analyses

To evaluate clinical efficacy in this setting, three groups of at least 20 subjects were needed. The differences in the three target parameter groups (Pycnogenol® or cranberry supplementation and control) in combination with SM and prophylaxis after 60 days was assessed. To assess the variations between the observational time before inclusion and the follow-up period in the 3 different groups, nonparametric statistics were applied. The analysis of variance (ANOVA) with Bonferroni correction or a Student's *t*-test was used to analyze the difference in means in the occurrence of UTI/R-UTI/IC in the 3 management groups, using Sigma Plot software [36]. Statistical significance was accepted at *p* < 0.05. Values are expressed as the number of patients or urines /(per) total number or as mean ± standard deviation (SD).

## 3. Results

Sixty-four subjects completed the registry study. The absence of a severe bacterial charge was verified with a urinalysis (according to the values in Table 1). Only patients without clinically significant bacterial charge were included in the study.

Table 2shows that the 3 groups of subjects (the two supplemented groups and the control group) that suffered from mild UTI/IC and that completed the study were demographically and clinically comparable.

No tolerability problem or side effect was observed in any supplement group.

Table 3shows the number of self-reported episodes of infection (as self-reported dysuria) in two months, 2 months before inclusion, and 2 months during follow-up.

The incidence of UTI symptoms during follow-up in comparison with the period before inclusion was significantly decreased with both supplements. In the SM group, the incidence also decreased significantly but to a lesser extent. In comparison, the rate of recurrent infections during Pycnogenol® supplementation was significantly lower than with cranberry supplementation (*p* < 0.05).

The number of infection-free subjects (as assessed by urinalysis) at the end of the two-month registry study was significantly higher with both supplements in comparison with SM (8/22) (*p* < 0.05) (Table 3). The number of infection-free subjects was significantly higher with Pycnogenol® (22/22) than with cranberry (7/20) (*p* < 0.05).

All subjects had significant symptoms of UTI or IC (minor pain, stranguria, repeated need for urination, lower, anterior abdominal pain) at inclusion.

The number of completely symptoms-free subjects after 2 months of management with Pycnogenol® (20/22) was significantly superior to standard management (18/22) and cranberry supplementation (16/20).

The occurrence of symptoms—all minor/mild—was broadly overlapping with the biochemical ‘normalization' of the urine.

Oxidative stress, as assessed by measuring the concentration of the plasma free radicals in Carr units, was high at inclusion in all subjects and was decreased at the end of the study with both supplements, Pycnogenol® having the most important effect (*p* < 0.05) (Table 2).

The association between decreased oxidative stress and reduction in UTIs and ICs may suggest a significant correlation between the two observations with important potential clinical applications.

## 4. Discussion

UTIs and ICs are common clinical entities often not arriving at the attention of the practitioner. Frequently, minor episodes are self-managed by patients with products usable without prescriptions [37]. Epidemiology of UTI indicates a common disease, apparently more frequent or severe in women and less severe, more tolerable in men [1, 4, 10, 38].

Most symptoms in recurrent UTIs or ICs are caused by persisting bacteriuria or persisting inflammatory processes in the lower urinary tract [39, 40]. Inflammation may persist due to the presence of bacterial fragments such as lipopolysaccharides (LPS), inducing an inflammatory response by triggering the release of cytokines [41, 42].

In this study, we investigated and compared the antioxidant and anti-inflammatory potential of the polyphenolic components in Pycnogenol® and cranberry for reducing the evolution and genesis of UTI, R-UTI, and IC. These polyphenolic compounds have been shown to reduce inflammation processes triggered by UTI or bacterial diseases in several studies [9, 11, 20, 22, 43].

It is important to consider that recurrent lower tract urinary infections (including interstitial cystitis) may have a preeminent nonbacterial component that may respond better to Pycnogenol® or cranberry than to antibiotics [14–17]. Inflammation, as indicated by symptoms, may persist well after bacteria are no longer present, as is thought to be the source for IC discomforts [40].

The anti-inflammatory effects of Pycnogenol® may be evident almost immediately by significantly reducing the symptoms. Cranberry is also known for its anti-inflammatory effects, but despite several studies, the exact mechanisms behind the UTI protection are still not clear yet [44–46]. Based on the results of this study, Pycnogenol® seems to have more important beneficial activity on UTI/IC-triggered inflammation. In previous *ex vivo* and *in vitro* studies, the potent anti-inflammatory effects of Pycnogenol® were widely investigated with the possible mechanism of action laying in the inhibition of NF-*κ*B and COX-1/COX-2 activation [47–50]. In addition, the oxidative stress levels at the end of the study were most significantly lowered with Pycnogenol®, compared to control and cranberry groups. Oxidative stress is strongly linked to inflammation, both being concurrently described in different pathological conditions and are interdependent [51]. Hence, the high impact of Pycnogenol® on the reduction of UTIs/IC in patients compared to the effect of cranberry in this study can partly be explained by the high antioxidative effect which is exerted by Pycnogenol®.

In addition to their anti-inflammatory and antioxidant activity, Pycnogenol® components or metabolites could decrease the virulence and diffusion of several bacterial strains including intestinal *E. coli,* as shown in several studies [20–24, 52–56]. The inflamed urothelial cells may cause protracted signs and symptoms and may also attract other bacteria restarting infections [56]. Based on previous findings, it can be suggested that components in Pycnogenol® and cranberry may have a special affinity for urothelial cells, reduce inflammation, and protect against bacterial adhesion and infections [20, 57–59].

With 75–90% of bacterial isolates, *Escherichia coli* (*E. coli*) is the most common uropathogen responsible for UTI and R-UTIs and was found particularly in women aged 25 to 44 years but is not necessarily the cause of infection [9, 38, 60–62].

*E. coli* can attach to uroepithelial cells with the help of their P-fimbriae via the activity of the P adhesin gene (papG) [60, 63]. The allele III variant has been associated with cystitis, but the connections between activity on bacteria and infection genesis are not yet fully established [60, 63]. There are hints that the high concentration of polyphenols present in natural supplements like cranberry and Pycnogenol® may inhibit the attachment of P-fimbriated *E. coli* to mucosal or bladder and uroepithelial cells in the lower urinary tract [9, 11, 20, 22, 43].

Since the main infectious bacteria responsible for UTI are *E. coli*, which are found in the gastrointestinal tract, this is another possible tissue in which active substances in cranberry and Pycnogenol® could reduce the infectiousness of the bacteria by interacting with them [38, 61].

Cranberry consumption is widely recommended for UTI prophylaxis. However, there is a debate in the literature whether the effects are significant [64]. Besides cranberry, there are more nonantibiotic treatments for UTI infections, such as D-Mannose, probiotics, methenamine hippurate, estrogens, or intravesical glycosaminoglycans [65–67]. Furthermore, there are even immunostimulants and vaccines that have been developed against UTI [65]. However, for most of these supplementary compounds and agents, there are not enough conclusive data to rely on, or to use them for the treatment of UTI/IC.

Possible limitations of this study were the relatively small number of subjects and the follow-up time of 2 months. Being a pilot registry supplement study, the data resulting from it can be seen as guidelines for further studies in this field. Furthermore, even after this relatively short amount of time, the results were statistically significant. Another limitation could be that the groups were not parallel and the UTI rate was compared with the medical history from previous infections. However, this procedure is common in pilot registry supplement studies, such as the present and has shown to be useful, providing interesting insights into important borderline conditions that are otherwise often overlooked in clinical practice.

Based on the results of this study, Pycnogenol®—with its high level of safety and tolerability–seems to effectively lower the recurrence of urinary tract infections with more important effects on inflammation and UTI/IC-symptoms than cranberry and can possibly be used as a prophylaxis. Of course, bigger studies with more patients and possibly with a longer follow-up time are needed to confirm the results of this pilot registry study.

## 5. Conclusion

This pilot registry indicates that 60 days of Pycnogenol® supplementation may decrease the occurrence or signs and symptoms related to the recurrence of UTIs and ICs without side effects and with good tolerability. Pycnogenol® compares well with cranberry, generally used in this condition. The effects of Pycnogenol® in these patients—including the control of oxidative stress—may be very important, particularly when a predominantly inflammatory component is present and continues the inflammatory process, like it is observed in UTI without infection, such as in IC or with a minimal bacterial component due to bacterial fragments.

## Figures and Tables

**Table 1:** Upper limits for defining biochemically “normal” urines according to the guidelines for urinalysis by Simerville et al. [13].

Component	Upper limit	Unit
Red blood cells (RBCs)/erythrocytes	2-3	Per high power field (HPF)
RBC casts	Negative	
White blood cells (WBCs)/leukocytes	Negative-10	Per *µ*l or mm^3^
Hemoglobin	Negative	Dipstick scale 0 to 4+
Bacterial culture	<100,000	colony forming units per milliliter (cfu/ml)

**Table 2:** Details of patients.

Properties		Controls + SM	Pycnogenol® + SM	Cranberry + SM
Cases		22	22	20
Females		14	12	13
Age ± SD		38.1 ± 2	38.3 ± 3.4	38 ± 2.2
Oxidative stress	Inclusion	379 ± 13	377 ± 21	383 ± 19
(Carr units ± SD)	Study end	370 ± 24	334 ± 19	366 ± 22

All subjects were under the standard management (SM).

**Table 3:** Number of self-reported episodes of UTI infection/inflammation in two months (before and during a 2-month follow-up).

Time	Controls (*n* = 22)		Pycnogenol® (*n* = 22)		Cranberry (*n* = 20)	
	2 months before inclusion	2 month-follow-up	2 months before inclusion	2 month-follow-up	2 months before inclusion	2 month-follow-up
Number of UTI episodes in 2 months	3.1 ± 0.2	2.2 ± 0.2	3.13 ± 0.3	1.2 ± 0.2^*∗*^	3.3 ± 0.4	1.8 ± 0.3
Time	At inclusion	After 2 months	At inclusion	After 2 months	At inclusion	After 2 months
Number of patients with ‘infection-free' urine^#^	9/22	8/22	11/22	22/22^*∗*^	10/20	7/20
Number of symptoms-free patients	0/22	18/22	0/22	20/22^*∗*^	0/20	16/20

Results before Pycnogenol® or Cranberry vs. during Pycnogenol® or Cranberry administration. The number of subjects who had “infection-free” urine and were symptom-free is shown for each group. ^*∗*^*p* < 0.05vs. controls and cranberry. ^*#*^Definition of “normal” urines as in Table 1[13].

## Data Availability

The data supporting the findings of this study are included within the article.
